# Comparing the diagnostic concordance of tele-EMS and on-site-EMS physicians in emergency medical services: a retrospective cohort study

**DOI:** 10.1038/s41598-020-75149-8

**Published:** 2020-10-22

**Authors:** Lina T. M. Quadflieg, Stefan K. Beckers, Sebastian Bergrath, Ann-Katrin Brockert, Hanna Schröder, Anja Sommer, Jörg C. Brokmann, Rolf Rossaint, Marc Felzen

**Affiliations:** 1 Department of Anaesthesiology, Medical Faculty, RWTH Aachen University, Aachen, Germany; 2 Department of Paediatrics, Klinikum Dortmund, Dortmund, Germany; 3 Aachen Fire Department, Aachen, Germany; 4 Emergency Department, Kliniken Maria Hilf Mönchengladbach, Mönchengladbach, Germany; 5 Faculty of Health, Medicine and Life Sciences, Care and Public Health Research Institute, Maastricht University, Maastricht, The Netherlands; 6 Emergency Department, University Hospital RWTH Aachen, Aachen, Germany

**Keywords:** Diseases, Health care, Outcomes research, Medical research, Preclinical research

## Abstract

In 2014, a telemedicine system was established in 24-h routine use in the emergency medical service (EMS) of the city of Aachen. This study tested whether the diagnostic concordance of the tele-EMS physician reaches the same diagnostic concordance as the on-site-EMS physician. The initial prehospital diagnoses were compared to the final hospital diagnoses. Data were recorded retrospectively from the physicians’ protocols as well as from the hospital administration system and compared. Also, all diagnostic misconcordance were analysed and reviewed in terms of logical content by two experts. There were no significant differences between the groups in terms of demographic data, such as age and gender, as well as regarding the hospital length of stay and mortality. There was no significant difference between the diagnostic concordance of the systems, except the diagnosis “epileptic seizure”. Instead, in these cases, “stroke” was the most frequently chosen diagnosis. The diagnostic misconcordance “stroke” is not associated with any risks to patients’ safety. Reasons for diagnostic misconcordance could be the short contact time to the patient during the teleconsultation, the lack of personal examination of the patient by the tele-EMS physician, and reversible symptoms that can mask the correct diagnosis.

## Introduction

Due to the demographic change, prehospital emergencies increase by 3.7% annually^1–4^. At the same time, there is a steadily increasing shortage of on-site-EMS physicians, especially in rural areas of Germany^5^. In order to counter this problem, the EMS needs to be optimised. Examples of optimisation are the introduction of a structured emergency call query by the dispatch center^6^, a new law for training of paramedics or the implementation of telemedicine in the EMS. Telemedicine in the EMS is currently being introduced in some regions in Germany^7,8^, the first holistic system was implemented in the city of Aachen, Germany in 2014^9^. The telemedicine system allows an immediate consultation with an EMS-physician at any time and, consequently, enables a higher quality of emergency medical care.

During the years after implementation, the number of telemedicine consultations for prehospital emergency patients increased. The tele-EMS physician can offer support for paramedics especially regarding diagnosis.

The diagnosis in the prehospital phase often determines the prehospital therapy and influences the choice of the hospital, if hospitalisation is needed. Therefore, it is important to know whether a distant tele-EMS physician can determine the correct diagnosis from the given information by the paramedics. As there are no predefined diagnoses for both, on-site-EMS and tele-EMS-physician, the tele-EMS physician needs to determine the correct diagnosis based on the information provided by the paramedics using clinical findings. The tele-EMS physician primarily supports the paramedics, but can also speak to the patient himself by phone if necessary. As the correct diagnosis lays the foundation for further treatment at hospital, it could be hypothesised that these diagnoses influence the prognosis and outcome of patients by preventing unnecessary delays to therapy initiation. Nevertheless, there are hardly any evaluations of the diagnostic concordance of on-site-EMS physicians and none of tele-EMS physicians^10^.

Therefore, this study aims to analyse whether the diagnoses of a tele-EMS physician is as precise as those of an on-site-EMS physician^11,12^.

## Methods

In this retrospective cohort study. Data were recorded from emergency protocols as well as from hospital information systems in order to compare prehospital diagnoses with the final diagnoses determined by the hospital.

### Patient data

Patients who were transported to the University Hospital RWTH Aachen were included in the study. Besides patients’ diagnoses, demographic data, such as age, gender, date of discharge, or death, were extracted from the prehospital protocols and the hospital administration system.

### Ethics vote

The local ethics committee granted an analysis of the data for quality assurance purposes and waived the requirement of informed consent (EK109/15, University Hospital RWTH Aachen). All data were recorded and saved anonymously.

### The technical set-up of the tele-EMS system and physician’s qualification

The Department of Anaesthesiology of the University Hospital RWTH Aachen developed from 2007 to 2013 a holistic telemedicine system as an addition to the conventional prehospital emergency system^13^. This system was implemented as an additional structure into the regular 24/7 routine EMS of Aachen in April 2014^14–16^. In the city of Aachen, there are twelve ambulances, two EMS-physicians, three primary care hospitals und an university hospital. Before the system was implemented, an EMS physician was involved in approximately 30% of all missions (22,055 in 2015), after the implementation the on-site EMS physician was involved in approximately 20% and the tele-EMS-physician in approximately 10% of all missions.

The two main components of the telemedicine system are the teleconsultation centre and the telemedically-equipped ambulances. A mobile communication unit is attached to the monitor-defibrillator-unit (Corpuls3; GS Elektromedizinische Geräte G Stemple, Kaufering, Germany) and allows data transmission from the following components: audio communication between paramedics and the tele-EMS physician; real-time vital data transmission; 12-lead ECG transmission; picture transmission from a connected smartphone; real-time video streaming from a 360° camera in the ambulance; printing of a teleconsultation report inside the ambulance; and position of the ambulance using GPS technology.

The teleconsultation centre consists of a 180° computer workstation with four standard monitors, on which the transmitted data are displayed using a specifically developed software named TM-Doku (Telemedical documentation; umlaut telehealthcare GmbH, Aachen, Germany).

The tele-EMS physician is, in comparison to the on-site-EMS physician, available immediately. The average consultation time at the beginning of the implementation, i.e. at the time of the presented study, was 11 min, currently it is 9 min. The average duration of missions supervised by on-site-EMS physicians is 43 min.

The professional qualification of all tele-EMS physicians is based on former experience as an on-site-EMS physician from at least 500 on-site emergency missions combined with further training in the telemedicine system, as well as in communication with the EMS teams. All tele-EMS physicians fulfil the standard of at least being in their 5th year of residency in anaesthesiology.

The tele-EMS physician can offer support for paramedics and can delegate the administration of drugs to achieve a better quality of care for patients, as the paramedics must not administer any medication by law. Furthermore, he can bridge the time of diagnosis and treatment for life-threatening diseases and injuries until the on-site-EMS physician arrives on the scene. The paramedics decide in accordance with the SOP (standard operating procedure) when to consult the tele-EMS physician and the paramedics can only take live-saving actions such as defibrillation without the delegation of a physician if there is acute risk to life. In future, paramedics will have more skills due to the Emergency Paramedic Act passed in 2014 and the associated expansion of training from 2 to 3 years^17^. Nevertheless the tele-EMS physician decides whether transportation to a hospital is necessary or not, since the physician is the responsible leader of the scene itself. The telemedicine system allows an immediate consultation with an EMS-physician at any time, consequently the teleconsultation eliminates the physician-free period and enables an immediate start of medical treatment.

As teleconsultation allows the tele-EMS physician to attend multiple emergency cases at the same time, the finite resourced of an on-site-EMS physician can be used wisely and called out only in life-threatening emergencies. Therefore, this function is more often available for patients who really need an on-site-EMS physician for invasive advanced life support measures.

### Data sources and data analysis

Tele-EMS physicians' data were evaluated from January to September 2015. To enable an evaluation of the same diagnostic collective, a control group of on-site-EMS physicians’ data was chosen from January to March 2014, a period before the introduction of the telemedicine system. For analysing a comparable number of tele-EMS and on-site EMS missions, 3 months were included for the on-site EMS-cases and 9 months for the telemedicine cases.

To analyse comparable collectives, the following exclusion criteria were applied: patients under the age of 18 years, suicidality, resuscitation, polytrauma, psychosis, forced admission, rampage threat, premature birth, transportation to a palliative ward and dislocation of a tracheostomy tube. These exclusion criteria were based on diagnoses which are not found to be suitable for teleconsultation, but suitable for an on-site-EMS physician. In case of CPR for example, the missions are not supervised by a tele-EMS physician as this time critical treatment leads no room for a tele-consultation. Consequently, it is necessary that an on-site-EMS physician is on scene.

The physician’s diagnoses were analysed based on the standardised tele-EMS and on-site-EMS physician protocols. Up to three initial diagnoses per case were recorded from the tele-EMS and on-site-EMS physician protocols. These were extracted from the designated fields for diagnoses as well as from the free text entries and, afterward, manually digitised by the study group. There is only one diagnosis written in the designated field, however, a maximum of two further diagnoses were extracted from the free text in order to be able to depict the diagnosis more holistically. This was only the case when clear differential diagnoses were defined by the on-site- and tele-EMS physicians.

The suspected diagnoses of the tele-EMS and on-site-EMS physicians were compared with the diagnoses, which were extracted from the administrative data of the University Hospital RWTH Aachen at the end of the stay in the hospital. Therefore, in a first step, in order to guarantee a blinded process, the tele-EMS physician and on-site-EMS physician diagnoses were manually coded according to the ICD-groups (International Statistical Classification of Diseases and Related Health Problems), whereas the diagnoses of the university hospital RWTH Aachen were found as a defined ICD-10-code and used as the reference diagnosis in this study^18^. Subsequently, the precise ICD-code of the University Hospital RWTH Aachen was compared to the ICD-codes of the diagnoses of the tele-EMS and on-site-EMS physicians.

After data collection and processing, the diagnoses made by the tele-EMS and on-site-EMS-physicians were analysed and compared. Additionally, tracer diagnoses, leaning on the consensus document on emergency care, "acute coronary syndrome" (ACS), "stroke", “cardiac diseases”, "hypertension", "epileptic seizure", and “chronic obstructive pulmonary disease” (COPD) were identified and analysed individually in relation to diagnostic miscordances. In addition, all diagnostic miscordances were analysed and reviewed in terms of logical content by two experts.

The use of the camera system, which is implemented in every ambulance, was also analysed^19^.

### Statistical methods

Statistical significance was tested using the Fisher exact test and the unpaired t-test using SPSS Statistics, Version 25.0 (IBM Corporation, Armonk, New York, USA). The probability of a type 1 error below 1% was considered as significant.

### Ethical approval

All authors confirm that all methods were carried out in accordance with relevant guidelines and regulations of the Declaration of Helsinki.

## Results

There were no significant differences between included patients treated by tele-EMS physicians and on-site-EMS physicians in terms of demographic data, such as age and gender, as well as regarding the hospital length of stay, as shown in Table 1. Furthermore, hospital mortality did not differ between the two groups, as shown in Table 2.

**Table 1:** Demographic data.

	Tele-EMS physician (n = 584)	On-site-EMS physician (n = 634)	p value
Age (in years)	64.6 (SD ± 19.9)	64.2 (SD ± 20.2)	0.712
Female	293 (50.2%)	301 (47.5%)	0.359
Male	291 (49.8%)	333 (52.5%)	0.359
Time of hospitalisation (in days)	6.7 (SD ± 12.0)	6.0 (SD ± 8.6)	0.195
Hospital mortality (number of patients)	25 (4.3%)	25 (3.9%)	0.775

This table shows demographic data as well as the time of hospitalisation and mortality from patients treated by the tele-EMS physician in comparison to patients, treated by the on-site-EMS physician. The probability of a type 1 error below 1% was considered as significant.

**Table 2:** Hospital mortality.

	Tele-EMS physician (n = 25)	On-site-EMS physician (n = 25)	p value
Time of hospitalisation until death (days)	10.7 (SD ± 11.5)	9.8 (SD ± 20.6)	0.848
Patients who died < 24 h	0 (0%)	6 (24%)	0.022
Patients who died without a diagnostic miscordance	19 (76.0%)	20 (80.0%)	1.000
Patients who died with a diagnostic miscordance	6 (24.0%)	5 (20.0%)	1.000
By that < 24 h	0 (0%)	2 (0.3%)	0.490

This table shows the overall hospital mortality and the hospital mortality from patients with and without diagnostic miscordances treated initially by the tele-EMS physician in comparison to patients with diagnostic miscordance treated initially by the on-site-EMS physician. The probability of a type 1 error below 1% was considered as significant.

### Diagnostic concordance

**Figure 1:**
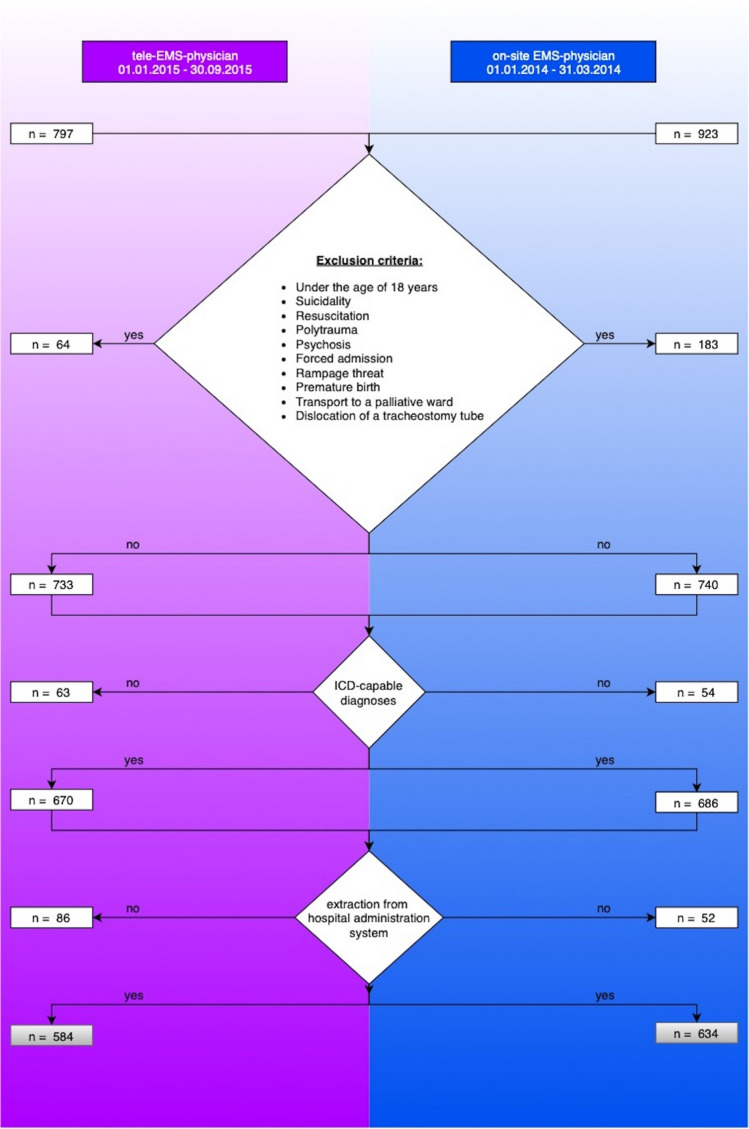
Selection of patient collective. This figure shows the in- and exclusion criteria of the patients treated by the tele-EMS physician (left side) and the on-site-EMS physician (right side) as well as the selection of the patient collective.

The prehospital diagnoses of 584 patients treated by the tele-EMS physician and 634 patients treated by the on-site-EMS physician were analysed. Due to the applied exclusion criteria, the cohorts and diagnoses are comparable in content and structure (Fig. 1).

After comparing the diagnoses using the ICD-10-system and after the double expert review, which compared the diagnoses content-wise and logically, there was no significant difference between the accuracy of the correct diagnoses in general (p = 0.0193). Consequently, the correct diagnosis was chosen in 82.0% (n = 520) and 76.5% (n = 447) of the patients treated by the on-site-EMS physician and tele-EMS physician, respectively.

**Figure 2:**
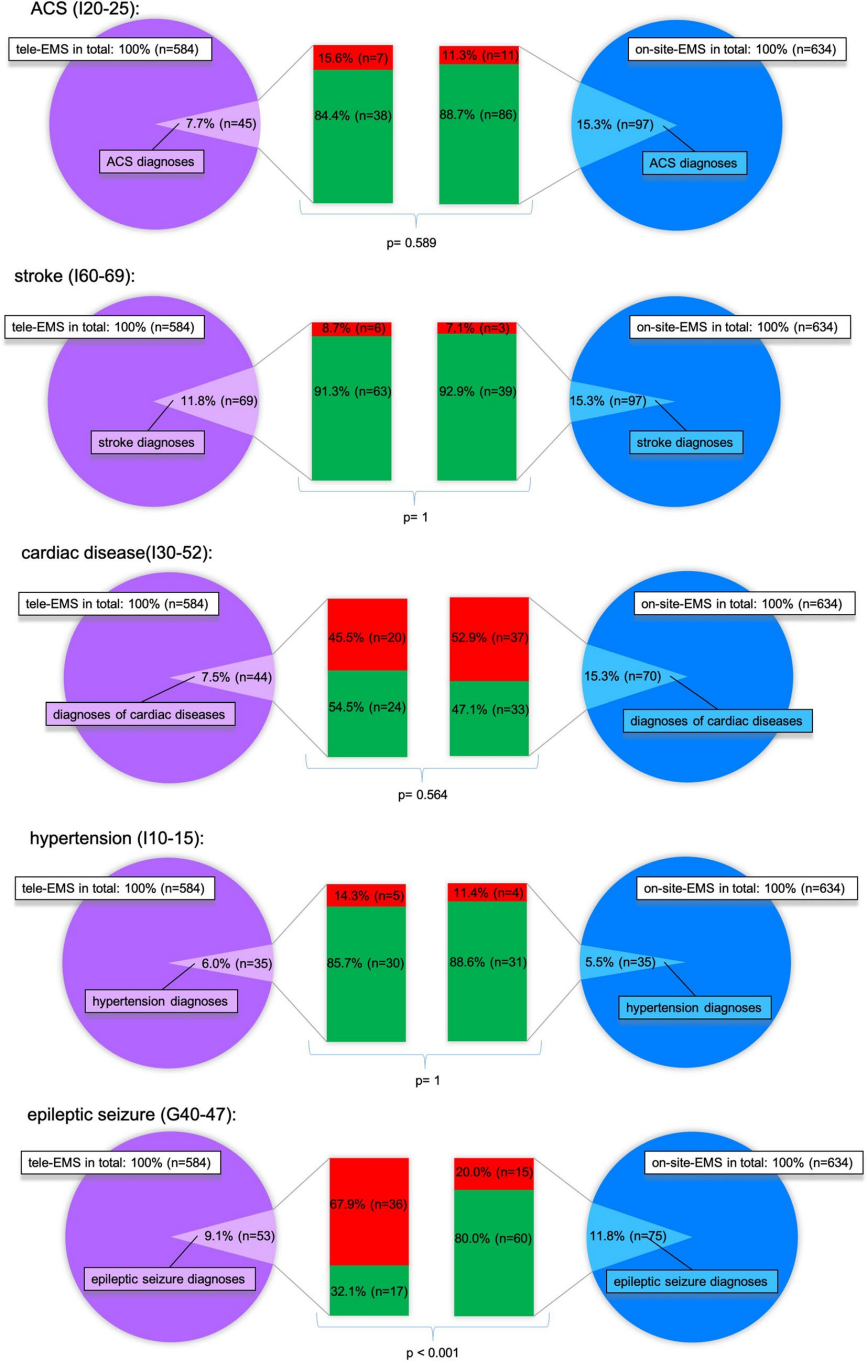
Diagnostic con- and miscordance. This figure shows matches (green bars) and fails (red bars) regarding diagnosis from the tele-EMS physician and the on-site-EMS physician compared with the final hospital diagnosis based on the hospital information system.

**Figure 3:**
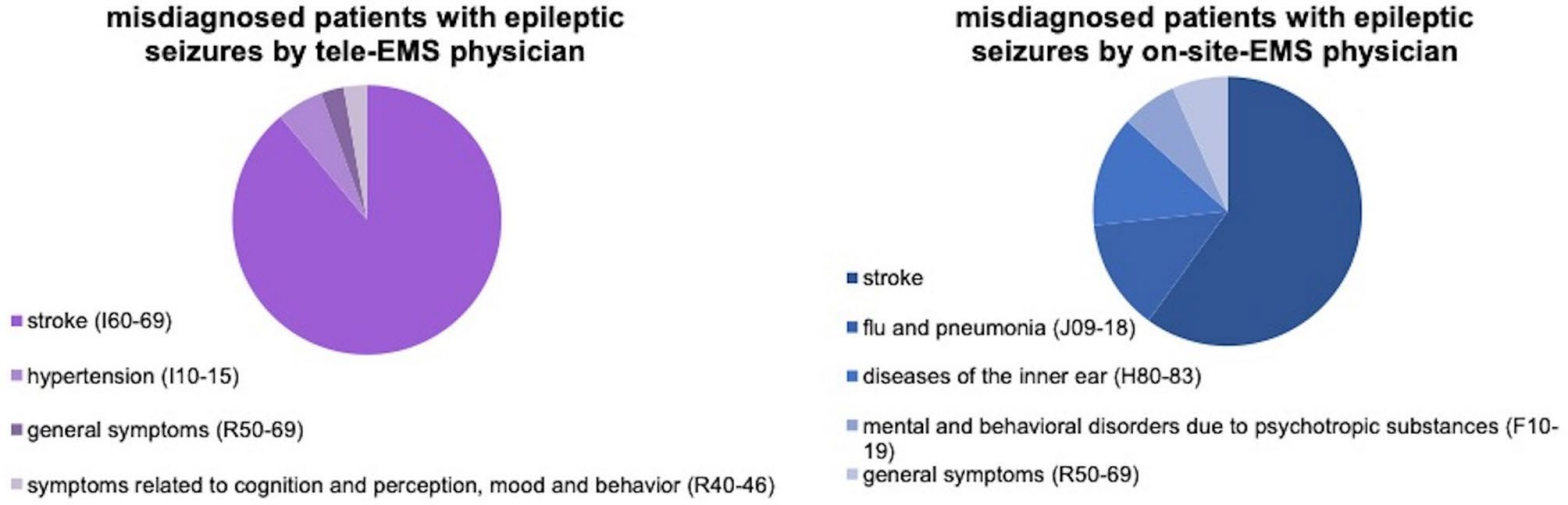
Misdiagnosed patients with epileptic seizures. This figure shows a comparison between tele-EMS physician and on-site-EMS physician regarding misdiagnosed patients with epileptic seizures.

As shown in Fig. 2, there were no significant differences in the comparison of the clinically important tracer diagnoses "ACS" (p = 0.589), "stroke" (p = 1), "hypertension" (p = 1), "cardiac disease" (p = 0.564) and "COPD" (p = 0.716), except for the diagnosis "epileptic seizure". The tele-EMS physician misdiagnosed the patients with epileptic seizures significantly more often than the on-site-EMS physician (p < 0.001). Figure 3 shows the alternative diagnoses of the misdiagnosed patients with epileptic seizures. The most often chosen incorrect diagnosis was “stroke”. This was the case in 88.9% (n = 32) of the seizure-patients (n = 36) treated by the tele-EMS physician and in 60.0% (n = 9) of the patients (n = 15) treated by the on-site-EMS physician.

### Death during hospitalisation after prehospital diagnostic misconcordances

Six patients receiving an initially incorrect diagnosis by the tele-EMS physician died during further treatment in the hospital. Looking closer at diagnoses and cause of death, the following was found: two patients were diagnosed with an ACS, of which one died of complications following a diaphragmatic hernia, and the other of pneumonia. The third patient was diagnosed with pneumonia and died due to heart failure. With these three patients, no correlation could be drawn in terms of the diagnoses and the cause of death. Diagnoses did not lead to a lack of therapy or prehospital intervention. The fourth patient, diagnosed with pneumonia, died of sepsis as a consequence of pneumonia. The fifth patient, who died of a urinary tract infection, was initially diagnosed with sepsis. The sixth patient was diagnosed with pulmonary edema and died because of an ACS. In those three cases, the causes of death showed a causal connection to the tele-EMS-diagnoses, even though they were not matching exactly in terms of wording.

Five patients who were wrongly diagnosed by the on-site-EMS physician also died during hospitalisation. Two of those died due to heart failure and were diagnosed by the on-site-EMS physician with pulmonary edema and tachycardia. The third patient was diagnosed with pneumonia and died of sepsis. The fourth patient died because of an open wound of the pelvis and was diagnosed with a femoral neck fracture. These four diagnoses can obviously be seen as directly related to the cause of death. The only diagnostic misconcordance with no correlation to the cause of death was a patient who died because of an acute renal failure and was diagnosed with COPD.

There was no significant difference between tele-EMS and on-site-EMS physicians in terms of duration between the time of admission and the time of death (10.7 ± 11.5 vs. 9.8 ± 20.6 days, p = 0.848). None of the deceased tele-EMS-patients and only two of the patients treated by the on-site-EMS physician died after less than 24 h of hospitalisation (p = 0.490). No significant differences could be observed in these patients in terms of gender (female: 4 vs 3; p = 1) and age (74.8 ± 16.7 vs. 75.0 ± 18.0 years; p = 0.016).

### Use of video streaming in the ambulance

In 54.6% (n = 319) of all teleconsultations (n = 584), information about the use of the video camera was found. Video streaming was used in 43.9% (n = 140) of those cases; in 56.1% (n = 179) video streaming was not used. Among the 36 cases where the tele-EMS physician misdiagnosed the patients with seizures, the camera was used in 25.0% (n = 9). In the cases where the tele-EMS physician correctly diagnosed those patients with epileptic seizures (n = 17), the camera was used in 35.3% (n = 6) of the cases. Altogether, the camera was not used significantly more often (p = 0.5196) on correctly diagnosed patients with seizures than on misdiagnosed patients.

## Discussion

This study aimed to investigate whether there is a difference in diagnosis quality between a tele-EMS physician and an on-site-EMS physician. Statistically higher accuracy of the one-site EMS-physicians could not be shown.

When comparing both systems, it was shown that the diagnostic concordance regarding tracer diagnoses does not differ significantly between tele-EMS physician and on-site-EMS physician, with the exception of the diagnosis "epileptic seizure". The mortality within 24 h after admission in the hospital does not differ significantly as shown in Table 2 (p = 0.022), the difference shown (0% vs. 24%) might exist because the tele-EMS physician does not treat patients with life-threatening conditions, while the on-site-EMS physician does.

This confirms the efficiency of telemedical therapy, which was already shown in 2016 by Brokmann et al. for ACS^20^. The rate of diagnostic misconcordance concerning “cardiac diseases” is high in both groups (54.5% vs. 52.9%) because of the multiple other diagnoses with similar symptoms and frequent combination of ACS, heart failure, lung edema, and tachycardia. In terms of COPD it is also high, as the diagnostic possibilities are very limited in the EMS. In the case of the epileptic seizure-patients, tele-EMS and on-site-EMS physicians both have high diagnostic misconcordance rates. In 88.9% (tele-EMS physicians) or 60.0% (on-site-EMS physicians) of those cases, the patients were diagnosed with "stroke", an important differential diagnosis of seizure. Due to the short time of preclinical treatment and the short time of patient contact, it is possible that reversible symptom complexes (e.g., Todd’s paralysis) were present during the prehospital phase but not in the hospital setting, thereby masking the correct diagnosis. Also, it can be assumed that the prioritised diagnosis of stroke (with a time-critical diagnostic significance) is preferably selected by tele-EMS or on-site-EMS physicians. A comparable study by Arntz et al. describes the underestimation of the severity of neurological disorders and discusses the use of support, such as, for example, video-assisted care for complex neurological diseases^21^. However, our data show that complex neurological conditions can be better understood by the on-site-EMS physician, who can observe and examine the patient personally. Therefore, we analysed the present data according to the use of video cameras, which was used in only about 25.0% of all teleconsultations. Most tele-EMS physicians think that the phone transfer of information via headset without the use of the video camera is sufficient. We detected no significant difference of misdiagnosed or correctly diagnosed patients with epileptic seizures. It should be mentioned that the video stream is only possible in the ambulance itself, whereas most of the treatment starts outside the ambulance. Moreover, as a consequence of this study in the standardised questionnaire of the tele-EMS physician, questions regarding epileptic seizures were implemented to be asked by the tele-EMS physician, which should lead to a higher correct diagnosis rate.

The correct prehospital diagnosis is particularly important when a special department is required for the treatment; for example catheterisation laboratory for ACS-patients or endovascular intervention for stroke-patients, which is not available in every basic and standard care provider. A diagnostic misconcordance with the selection of the wrong hospital can consequently lead to a possibly life-threatening therapy delay.

Prehospital diagnostic options are limited (compared to intrahospital diagnostic possibilities) due to restricted apparatus and laboratory diagnostic features. Furthermore, working under time pressure, on-scene might also limit the prehospital diagnosis. Thus, the tele-EMS physician, who is, in comparison to the on-site-EMS physician, available immediately, has an advantage of being shielded from the emergency scenery and, therefore, can apply maximum concentration. He can also easily use the provided SOP and review the latest recommendations of various medical societies and, therefore, work based on up-to-date algorithms. In order to come to the right diagnosis, the tele-EMS physician is highly dependent on accurate information from the paramedics’ team on-site, whereas the on-site-EMS physician has the possibility to examine and consult the patient himself. Patients with respiratory problems for example are very hard to examine as the tele-EMS physician needs to trust the paramedic on information about breath sound, speed and depth. Yet, it has to be said, that diagnostic mistakes occur in prehospital as well as in intrahospital settings^22^.

Studies on stroke-patients show the reliability and accuracy of telemedical support. It provides a faster access to specialised treatment and accelerates the door-to-needle-time^23,24^. The establishment of clinical prediction rules helps the paramedics classify patients with strokes in order to receive endovascular intervention as fast as possible. These studies rather investigate the time-opimised patient care in isolation than considering differential diagnoses^25^.

According to the emergency paramedic law, a training curriculum could be helpful. A standardised and structured procedure could lead to an improvement of taking the patients’ medical history and consequently improve the transfer of information to the tele-EMS physician. Structured basic education, training, and further education of tele-EMS and on-site-EMS physicians are critical to achieving a high diagnostic concordance^26–28^.

Since the prehospital care of the patients usually ends with admission to the appropriate hospital, a feedback system would be required, similar to the response number of the EMS in the province of Hessen^29,30^. Such a system could inform the tele-EMS physicians and on-site-EMS physicians, as well as the EMS paramedics, about the final diagnoses and thus help to verify the initial diagnoses, thus providing an opportunity for subjective evaluation of possible diagnostic errors^21^. This could, consequently, serve as a regular, personal re-evaluation. Likewise, a correct diagnosis can be confirmed and also be regarded as a personal learning process that could be transferred to future cases by the attending physician.

In conclusion, it could be shown that the ICD-codes are not an optimal classification for comparing diagnoses; especially not for preclinical diagnoses that are not already available as an ICD-code. Because there are several ICD-codes for the same diagnosis, this procedure can easily lead to errors. The results are still very meaningful and significant despite the different presentation of the diagnoses (free text vs. ICD-Code), as this leads rather to a shift of the results at the expense of diagnostic misconcordance. However, another type of comparison should be considered in future research. Any other method would be expected to give better results in terms of diagnostic concordance.

There are a few limitations.

Only patients admitted to the university hospital RWTH Aachen were included in the study because analysing the data of the three other hospitals in Aachen was not possible due to privacy policies, but more than 50% of all patients are transported to the university hospital RWTH Aachen, which is the only hospital in Aachen with a department for neurology and a cardiac catheterisation laboratory.

Secondary transports were not investigated; these will be evaluated in further studies. This may reveal patients who were initially transported to the wrong hospital. Missions where the tele-EMS physician was bridging the time until an on-site-EMS physician was on scene were also not investigated. An on-site-EMS physician from neighbouring areas, when no other on-site-EMS is available in the city of Aachen, is required about 30 times a month. They take about 10 min longer to be on scene. In these cases the TNA can bridge the gap. Those missions will also be investigated in further studies.

The telemedical and emergency medical diagnoses were coded by diagnostic keywords in ICD-groups. By contrast, the diagnoses documented in the hospital administration system were available as a precisely defined ICD-code. It was shown that a tele-EMS physician’s or on-site-EMS physician’s diagnosis was considered wrong, but instead was identical to the diagnosis documented in the hospital administration system, as the ICD-groups are broader and several diseases of one symptom complex are summarised, as well as the fact that there exist several ICD-codes for one and the same diagnosis.

Due to the handwritten recording of patient data, transmission errors when filling out the application protocols and when manually entering the protocol data into a digital table cannot be ruled out and might be the cause of missing or incomplete patient data, which then led to the exclusion of those patients.

In addition, the missions without a physician involved were not evaluated because an EMS physician, on-site-EMS or tele-EMS physician, must always be involved in every potentially life-threatening emergency. This is laid down by law, since paramedics in Germany are not allowed to administer drugs. Accordingly, a physician is always involved when medical therapy is required.

The rare use of the video camera in those cases with patients with seizures must also be mentioned.

It has to mentioned, that in spite of the many obvious advantages of telemedicine in general, the implementation of a telemedical system in all areas of medical care is hardly realisable due to costs, technical implementation, technical know-how and data protection regulations as privacy policies. Thus its generalisation can be limited.

## Conclusion

No significant differences in patient outcomes in terms of length of stay, morbidity and mortality could be found in this study. It was shown that the diagnostic concordance (in general and regarding tracer diagnoses) does not differ significantly between tele-EMS and on-site-EMS physicians, with the exception of the diagnosis "epileptic seizure". Regarding the diagnosis of "epileptic seizure", both the tele-EMS physician and on-site-EMS physician often made the mistaken diagnosis of "stroke", which does not contribute any added risk to patient.

To avoid diagnostic misconcordance in cases of neurological diseases, it might be helpful to make more frequent use video-assisted aids to help the tele-EMS physician to examine the patient even more systematically and holistically. As a consequence of these findings, the questionnaire in case of assumes strokes, posed by the tele-EMS physicians to the ambulance staff, has been extended by the question, whereas the anamnesis shows any signs for epileptic seizures or anticonvulsant medication. As a consequence of the presented results, however, the procedural instructions were changed, so that the use of video is mandatory for all patients with neurological conditions, as far as they agree.

Future investigations will be needed to show whether the video consultation and the extension of the questionnaire lead to a further improvement of the diagnostic concordance^13^.

In summary, this study shows that telemedicine is definitely relevant to the future prehospital medical care and that a patient-oriented and highly qualified EMS can no longer be imagined without telemedical support.
